# THE HEALTH OF THE DIRECT CARE WORKFORCE: EVIDENCE AND IMPLICATIONS ACROSS LONG-TERM CARE

**DOI:** 10.1093/geroni/igac059.624

**Published:** 2022-12-20

**Authors:** Kezia Scales, Kezia Scales

## Abstract

Direct care workers play a key role in supporting the health and wellbeing of older adults and people with disabilities across care settings—yet their own health risks are largely overlooked. The four papers in this symposium address this critical knowledge gap. First, McCall and colleagues will present a comparative analysis of the health status, health insurance coverage, and healthcare experiences of direct care workers across long-term care using National Health Interview Survey data. Next, Lee et al. will present the trends and characteristics of occupational injuries and illnesses among California’s long-term care workers from 2019 to 2020 using California Workers’ Compensation data, assessing the impact of COVID-19 on their occupational health. Sterling will characterize the physical and mental health of the direct care workforce before and during COVID-19 using data from the CDC's Behavioral Risk Factor Surveillance Survey, as well as drawing on qualitative and survey-based studies of unionized, agency-employed home care workers in New York. Sterling will also present findings from a pilot feasibility study of an intervention aimed at improving home care workers’ well-being. Finally, Quinn et al. will synthesize findings on home care workers’ occupational hazards—including needlesticks, musculoskeletal strain, violence and infections—and examine how preventing these risks can improve safety for both workers and clients. The discussant will draw out themes and implications from across these complementary studies, highlighting the importance of safeguarding direct care workers’ health as a key step toward improving care quality and outcomes for older adults and people with disabilities.

